# Duckweeds as Multiplexable Plant Models for Exploring Abiotic Stress Responses

**DOI:** 10.64898/2026.09.03.749270

**Published:** 2026-09-07

**Authors:** Bryan Ramirez-Corona, Santiago Cadena, Kerry Bubb, Christine Queitsch, Josh T. Cuperus

## Abstract

In nature, plants experience complex combinations of environmental stresses. Prior studies using *Arabidopsis thaliana* show that transcriptional responses to combinatorial stress are unique and cannot be predicted from responses to individual treatments, making it imperative to study combinatorial stress in the monocotyledonous cereals that comprise the majority of our food supply. However, crop species are not easily amenable for such studies because of their large size, long generation time, and organismal complexity. In contrast, the monocotyledonous aquatic duckweeds are small, grow rapidly, and show reduced organismal and genomic complexity. Here, we explore the physiological responses to combinatorial stress in three duckweed species, *S. polyrhiza* , *L. minor* , and *W. australiana* and examine their potential to serve as multiplexable models. Focusing on *S. polyrhiza,* the most complex and best annotated species, we investigated transcriptional response to individual and combinatorial stress treatments. While most individual treatments elicit few transcriptional changes, combinatorial stress elicited a non-additive transcriptional response unique to each combination. Exploring the duckweed regulatory landscape, we found that regulatory regions near genes that were differentially expressed in combinatorial stress in *S. polyrhiza* resemble environmentally responsive regulatory elements found in terrestrial plants. Examined duckweed species show numbers and genomic distributions of regulatory elements similar to those of *A. thaliana* and maize, with subtle effects of genome size but none of organismal complexity. Taken together, our results establish that duckweeds respond to combinatorial stress in a manner similar to terrestrial plants and can therefore serve as high-throughput, multiplexable models for studying its molecular underpinnings.

## Full Text Availability

The license terms selected by the author(s) for this preprint version do not permit archiving in PMC. The full text is available from the preprint server.

